# Supplementary material for: Maternal Supplementation of Food Ingredient (Prebiotic) or Food Contaminant (Mycotoxin) Influences Mucosal Immune System in Piglets
Source: Nutrients. 2020 Jul 17;12(7):2115. doi: 10.3390/nu12072115 (PMC7400953; doi:10.3390/nu12072115)
Supplement: Supplementary file 1 [file nutrients-12-02115-s001.zip › Table S1.pdf]

Table S1: Diet composition. Diets were formulated according to the nutrient and energy requirements of gestating and lactating sows (Cooperl, Lamballe, France) and supplemented with either maltodextrin as a control or PREB or DON. As usual, piglets received started first phase from weaning (PND28) during 7-10 days, fed started second phase until they weighed less than 25 kg (PND49) and finally fed with standard (STD) grower (PND90). [1]CTRL: control diet; PREB: maternal diet supplemented with scFOS (Profeed®); DON: maternal diet supplemented with DON. [2]The premix (Cooperl, Lamballe, France) supplied per kg of diet: retinol 10000 UI, cholecalciferol 1500 UI, alpha-tocopherol 45 mg, menadione 2 mg, thiamine 2 mg, riboflavin 4 mg, niacin 20 mg, D-pantothenic acid 10.9 mg, pyridoxine 3 mg, D-biotin 0.2 mg, folic acid 3 mg, vitamin B12 20 µg, choline 500 mg, Fe 81.5 mg as ferrous carbonate and sulphate, Cu 10 mg as copper sulphate, Mn 40 mg as manganese oxide, Zn 99.2 mg as zinc oxide, Co 0.1 mg as cobalt carbonate, I 0.6 mg and Se 0.3 mg

	CTRL[1]		PREB[1]		DON[1]		starter (1st phase)	starter (2nd phase)	STD grower
	Gestation	Lactation	Gestation	Lactation	Gestation	Lactation			
**Ingredients (g.kg^{-1} fresh matter)**									
Wheat	229	256	229	256	229	256	-	232	262
Corn	110	120	110	120	110	120	-	250	160
Barley	348	257	348	257	348	257	453	241	255.5
Wheat bran	159	100	159	100	159	100	-	-	50
Soybean meal	100	180	100	180	100	180	175	226	190
Lard	-	-	-	-	-	-	-	-	-
Soybean protein	-	-	-	-	-	-	25	-	-
Dehydrated whey	-	-	-	-	-	-	200	-	-
Refatted skim-milk	-	-	-	-	-	-	80	-	-
Sugarcane molasse	-	30	-	30	-	30	-	-	30
Palm oil	20	20	20	20	20	20	23	5.0	20
Maltodextrin	3.3	1.5	-	-	3.3	1.5	-	-	-
scFOS	-	-	3.3	1.5	-	-	-	-	-
DON	-	-	-	-	0.003	-	-	-	-
Calcium carbonate	17.3	12.0	17.3	12.0	17.3	12.0	14.1	11.3	12.9
Bicalcium phosphate	3.0	10.2	3.0	10.2	3.0	10.2	-	-	5
Sodium chloride	4.5	4.5	4.5	4.5	4.5	4.5	-	4.0	4.5
Trace element and vitamin mix[2]	5.0	5.0	5.0	5.0	5.0	5.0	5.0	5.0	6.1

**Composition (g/100g)**		
Minerals	5.7	6.1
Protein	13.4	16.4
Fat	4.1	4.2
Fibre	4.4	4.1
Starch	42.8	38.9
