# Supplementary material for: Maternal Supplementation of Food Ingredient (Prebiotic) or Food Contaminant (Mycotoxin) Influences Mucosal Immune System in Piglets
Source: Nutrients. 2020 Jul 17;12(7):2115. doi: 10.3390/nu12072115 (PMC7400953; doi:10.3390/nu12072115)
Supplement: Supplementary file 1 [file nutrients-12-02115-s001.zip › Table S2.pdf]

Table S2: Cytokines used for ELISA and/or qRT-PCR. Studied cytokines or others factors (everyone was tested by qRT-PCR, some only for ELISA) grouped accordingly of their main function in immunity, with their main cell sources and their specific functions in immunity for each ones.

FUNCTIONS	CYTOKINES / FACTORS
Onset and maintenance of inflammatory/innate response	IL-1β, IL-6, IL-8, IL-12p40, IL-23A, TNFα
Specific Immunity regulation	***DRIVING CYTOKINES***
	Th1 driving cytokines: IL-12p40
	Th17 driving cytokines: IL-6, IL-23A
	EFFECTOR CYTOKINES
	Th1 cytokines: IL-2p40, IFNγ
	Th17 cytokines: IL-17
	Treg: IL-10, TGFβ
Homing, inflammatory/immune cell recruitment	Chemokines (IL-8, CCL20, CX3CL1)
Control and switching off inflammation and immunity	IL-10, TGFβ, FoxP3
Proliferation and differentiation of B cells	BAFF
Toll-like receptors	TLR4, TLR5

CYTOKINES	MAIN CELL SOURCES	FUNCTIONS
IL-1β	Epithelial cells, infected cells, neutrophils, macrophages	Activation of inflammatory/innate cells and endothelial cells; fever induction
IL-6	Endothelial cells, neutrophils, macrophage	Activation of inflammatory/innate cells and endothelial cells; endocrine effects
IL-8	Epithelial, endothelial and innate cells	Recruitment and activation of neutrophils
IL-12p40	Dendritic cells, macrophages	Th1 induction
IL-17A	Th17 $CD4^+$ lymphocytes	Pro-inflammatory activity; recruitment and activation of neutrophils; increase of antimicrobial peptides
IL-23A	Dendritic cells, macrophages	Activation of inflammatory/innate cells and endothelial cells, Th17 $CD4^+$ lymphocytes
IFNγ	NK cells, γ/δ T lymphocytes, Th1 $CD4^+$ lymphocytes, cytotoxic T CD8+ lymphocytes	Major role in the cell-mediated immunity against intracellular pathogen: activation of macrophages, NK cells, CTL, maintenance of Th1 differentiation.
TNFα	Epithelial cells, infected cells, neutrophil, macrophage, NK cells	Activation of inflammatory/innate cells and endothelial cells; endocrine effect; catabolic induction
IL-10	M2 polarized macrophages, Treg lymphocytes, Th2 $CD4^+$ lymphocytes	Anti-inflammatory and immunosuppression activity
TGFβ	Treg lymphocytes, Th2 $CD4^+$ lymphocytes	Anti-inflammatory and immunosuppression activity
