# Supplementary material for: Maternal Supplementation of Food Ingredient (Prebiotic) or Food Contaminant (Mycotoxin) Influences Mucosal Immune System in Piglets
Source: Nutrients. 2020 Jul 17;12(7):2115. doi: 10.3390/nu12072115 (PMC7400953; doi:10.3390/nu12072115)
Supplement: Supplementary file 1 [file nutrients-12-02115-s001.zip › Table S3.pdf]

Table S3: Primers used for quantitative RT-PCR. Cyclophilin A and RPL32 genes were used as housekeeping genes. qRT-PCR was used to monitor migration of immune cells (CCL20, CX3CL1), intestinal tolerance (FoxP3, IL-10, TGFβ), inflammation and/or specific immunity regulation (IFNγ, IL-1β, IL-6, IL-12p40, IL-17A, IL-23A) as well as expression of gene involved in IgA production (BAFF) and TLR4 or TLR5 that could be down- or up-regulated following the different diet of sows and/or piglets.

Gene Symbol	Gene name	Primer sequence	References
Cyclo A	Cyclophilin A	F: CCCACCGTCTTCTTCGACAT<br>R: TCTGCTGTCTTTGGAACTTTGTCT	MN_214353
RPL32	Ribosomal Protein L32	F: AGTTCATCCGGCACCAGTCA<br>R: GAACCTTCTCCGCACCCTGT	MN_001001636<br>[37]
BAFF	B-cell activating factor	F: GAGAGCAGCTCCATTCAAAG<br>R: GCATGCCACTGTCTGCAATC	[36]
CCL20	Chemokine (C-C motif) ligand 20	F: GCTCCTGGCTGCTTTGATGTC<br>R: CATTGGCGAGCTGCTGTGTG	NM_001024589<br>[36]
CX3CL1/Fractalkine	Chemokine (C-X3-C motif) ligand 1 or Fractalkine	F: GCAGCTCCTAGTCCATTAC<br>R: CACCATTCTGACCCAAAG	EST CK464144<br>[36]
FoxP3	Forkhead box P3	F: GGTGCAGTCTCTCTGGAACAA<br>R: GGTGCCAGTGGCTACAATAC	AY669812<br>[36]
IFNγ	Interferon gamma	F: TGGTAGCTCTGGGAAACTGAATG<br>R: GGCTTTGCGCTGGATCTG	NM_213948<br>[36]
IL-1β	Interleukin 1 beta	F: GAGCTGAAGGCTCTCCACCTC<br>R: ATCGCTGTCATCTCCTTGCAC	NM_001005149
IL-6	Interleukin 6	F: GGCAAAAGGGAAAGAATCCAG<br>R: CGTTCTGTGACTGCAGCTTATCC	NM_214399
IL-10	Interleukin 10	F: GGCCCAGTGAAGAGTTTCTTTC<br>R: CAACAAGTCGCCCATCTGGT	NM_214041
IL-12p40	Interleukin 12 p40	F: GGTTTCAGACCCGACGAACTCT<br>R: CATATGGCCACAATGGGAGATG	NM_214013
IL-17A	Interleukin 17 A	F: CCAGACGGCCCTCAGATTAC<br>R: CACTTGGCCTCCCAGATCAC	AB102693<br>[35]
IL-23A	Interleukin 23 A	F: GAGAAGAGGGAGATGATGAGACTACA<br>R: GGTGGATCCTTTGCAAGCA	[36]
TGFβ	Transforming growth factor beta	F: GAAGCGCATCGAGGCCATTC<br>R: GGCTCCGGTTCGACACTTTC	X54859<br>[35]
TLR4	Toll like receptor 4	F: GCCATCGCTGCTAACATCATC<br>R: CTCATACTCAAAGATACACCATCGG	[36]
TLR5	Toll like receptor 5	F: CCTTCCTGCTTCTTTGATGG<br>R: CTGTGACCGTCCTGATGTAG	[36]
