# Supplementary material for: Maternal Supplementation of Food Ingredient (Prebiotic) or Food Contaminant (Mycotoxin) Influences Mucosal Immune System in Piglets
Source: Nutrients. 2020 Jul 17;12(7):2115. doi: 10.3390/nu12072115 (PMC7400953; doi:10.3390/nu12072115)
Supplement: Supplementary file 1 [file nutrients-12-02115-s001.zip › Table S4.pdf]

Table S4: Sow performance. In the twelve hours following farrowing, we recorded the litter size and individual piglet birth weights as well as their mortality rate before the first or second days of life of each group of piglets (litter of sows CTRL, PREB and DON). Non-parametric dataset were analysed using the Kruskal-Wallis test (1-way ANOVA).

	CTRL	PREB	DON	p
litter size (nb piglets)	15.00 ± 1.78	13.25 ± 2.46	13.75 ± 0.63	ns
birth weight (kg)	1.40 ± 0.06	1.25 ± 0.06	1.37 ± 0.05	ns
% piglet death < 24hrs	10.92 ± 5.98	18.55 ± 7.73	9.11 ± 1.76	ns
%piglet death < 48hrs	14.25 ± 4.76	21.65 ± 8.61	12.86 ± 2.03	ns
